# Investigating the variability in pressure–volume relationships during hemorrhage and aortic occlusion

**DOI:** 10.3389/fcvm.2023.1171904

**Published:** 2023-08-23

**Authors:** Fahim Usshihab Mobin, Antonio C. Renaldo, Enrique Carrasco Perez, James E. Jordan, Lucas P. Neff, Timothy K. Williams, M. Austin Johnson, Elaheh Rahbar

**Affiliations:** ^1^ Department of Biomedical Engineering, Wake Forest University School of Medicine, Winston Salem, NC, United States; ^2^ Virginia Tech, Wake Forest University School of Biomedical Engineering and Sciences, Blacksburg, VA, United States; ^3^ Advanced Computational Cardiovascular Lab for Trauma, Hemorrhagic Shock & Critical Care, Wake Forest University School of Medicine, Winston Salem, NC, United States; ^4^ Department of Cardiothoracic Surgery, Wake Forest University School of Medicine, Winston Salem, NC, United States; ^5^ Department of General Surgery, Section of Pediatric Surgery, Wake Forest University School of Medicine, Winston Salem, NC, United States; ^6^ Certus Critical Care™ Inc., Salt Lake City, UT, United States; ^7^ Department of Vascular and Endovascular Surgery, Wake Forest University School of Medicine, Winston Salem, NC, United States; ^8^ Department of Surgery, Division of Emergency Medicine, The University of Utah, Salt Lake City, UT, United States

**Keywords:** pressure–volume, cardiac function, longitudinal, hemorrhage, variability, REBOA, EVAC

## Abstract

**Introduction:**

The pressure–volume (P-V) relationships of the left ventricle are the classical benchmark for studying cardiac mechanics and pumping function. Perturbations in the P-V relationship (or P-V loop) can be informative and guide the management of heart failure, hypovolemia, and aortic occlusion. Traditionally, P-V loop analyses have been limited to a single-beat P-V loop or an average of consecutive P-V loops (e.g., 10 cardiac cycles). While there are several algorithms to obtain single-beat estimations of the end-systolic and end-diastolic pressure–volume relations (i.e., ESPVR and EDPVR, respectively), there remains a need to better evaluate the variations in P-V relationships longitudinally over time. This is particularly important when studying acute and transient hemodynamic and cardiac events, such as active hemorrhage or aortic occlusion. In this study, we aim to investigate the variability in P-V relationships during hemorrhagic shock and aortic occlusion, by leveraging on a previously published porcine hemorrhage model.

**Methods:**

Briefly, swine were instrumented with a P-V catheter in the left ventricle of the heart and underwent a 25% total blood volume hemorrhage over 30 min, followed by either Zone 1 complete aortic occlusion (i.e., REBOA), Zone 1 endovascular variable aortic control (EVAC), or no occlusion as a control, for 45 min. Preload-independent metrics of cardiac performance were obtained at predetermined time points by performing inferior vena cava occlusion during a ventilatory pause. Continuous P-V loop data and other hemodynamic flow and pressure measurements were collected in real-time using a multi-channel data acquisition system.

**Results:**

We developed a custom algorithm to quantify the time-dependent variance in both load-dependent and independent cardiac parameters from each P-V loop. As expected, all pigs displayed a significant decrease in the end-systolic pressures and volumes (i.e., ESP, ESV) after hemorrhage. The variability in response to hemorrhage was consistent across all three groups. However, upon introduction of REBOA, we observed significantly high levels of variability in both load-dependent and independent cardiac metrics such as ESP, ESV, and the slope of ESPVR (*E_es_*). For instance, pigs receiving REBOA experienced a 342% increase in ESP from hemorrhage, while pigs receiving EVAC experienced only a 188% increase. The level of variability within the EVAC group was consistently less than that of the REBOA group, which suggests that the EVAC group may be more supportive of maintaining healthier cardiac performance than complete occlusion with REBOA.

**Discussion:**

In conclusion, we successfully developed a novel algorithm to reliably quantify the single-beat and longitudinal P-V relations during hemorrhage and aortic occlusion. As expected, hemorrhage resulted in smaller P-V loops, reflective of decreased preload and afterload conditions; however, the cardiac output and heart rate were preserved. The use of REBOA and EVAC for 44 min resulted in the restoration of baseline afterload and preload conditions, but often REBOA exceeded baseline pressure conditions to an alarming level. The level of variability in response to REBOA was significant and could be potentially associated to cardiac injury. By quantifying each P-V loop, we were able to capture the variability in all P-V loops, including those that were irregular in shape and believe that this can help us identify critical time points associated with declining cardiac performance during hemorrhage and REBOA use.

## Introduction

Hemorrhage is the leading cause of potentially preventable deaths in trauma, causing approximately 91% of military and 35% of civilian fatalities (1, 2). In particular, non-compressible truncal hemorrhage (NCTH) is the leading cause of such deaths occurring prior to arrival at a treatment center, warranting the need for quick hemorrhage control (3). To address this problem, minimally invasive endovascular hemorrhage control (EHC) devices have been proposed as a lifesaving intervention to minimize blood loss and prolong survival.

An emerging EHC strategy is resuscitative endovascular balloon of the aorta (REBOA), which uses a balloon catheter to create a complete occlusion of the aorta. This procedure has become increasingly adopted as a minimally invasive clinical intervention to manage NCTH prior to a definitive treatment. The rise in popularity is due in large part to its capability to stop hemorrhage by restricting blood flow distal to the balloon while simultaneously augmenting proximal perfusion. Despite its success in restoring perfusion to vital organs such as the heart and brain, the adoption of REBOA has been limited due to its downstream ischemic burden (4, 5). Pre-clinical large animal studies suggest that Zone 1 REBOA (proximal to the diaphragm) is survivable for up to 60 min and Zone 3 (distal to renal artery) for 90 min. In humans, however, Zone 1 REBOA has been consistently lethal at 45 min (6–9).

To overcome this ischemic burden, current research is focused on alternative EHC or REBOA-like strategies. This has led to the development of partial aortic occlusion, including endovascular variable aortic control (EVAC) (10–12). EVAC utilizes a predefined algorithm to carefully titrate distal blood flow past the balloon, both off-loading supraphysiologic proximal blood pressure and minimizing ischemia by allowing for attenuated flow to distal tissue beds (13). Across several translational studies, partial aortic occlusion strategies demonstrate the capability to reduce distal organ injury while minimizing the rate of bleeding (14, 15). While there is a mounting evidence in support of EVAC and other partial REBOA strategies in response to hemorrhage are promising, their impact on cardiac function is not completely understood.

A handful of investigators have reported some findings related to myocardial injury and/or cardiac output (CO) changes during hemorrhage and aortic occlusion. For example, in 2019, Beyer et al. (16) reported a significant myocardial injury in pigs receiving REBOA after a 25% total blood volume hemorrhage. More recently, Edwards et al. (17) reported greater coronary perfusion in pigs who received a partial aortic occlusion in comparison with those who received complete occlusion with REBOA after a 25% hemorrhage. Clinically, Wasicek et al. (18) characterized the hemodynamic trends before, during, and after a Zone 1 or Zone 3 REBOA and reported that both groups experienced elevated heart rates (HRs), but significantly more patients experienced periods of hypertension (>140 mmHg) with a Zone 1 occlusion. In addition, they found that both groups experienced more hemodynamic variability upon balloon deflation than inflation. While these studies have illustrated some of the metrics of cardiac performance, there is a need to better understand the relationship between REBOA and EVAC and cardiac performance. There are very few studies that take a deep dive into characterizing the cardiac function during hemorrhage and aortic occlusion quantitatively. We suspect that this may be in part due to the challenges and complexity of analyzing longitudinal pressure–volume (P-V) loops during acute transient phases.

Classically, the left ventricle P-V loops and their derived indices have been the gold standard for quantifying cardiac function parameters. P-V loops are often analyzed using proprietary software with a focus on a simplified single-beat analysis of parameters. In response to this limitation, independent algorithms have emerged to perform both single-beat analysis and analyses over a range of P-V loops (19, 20). Recently, Stonko et al. (21) developed an open-source MATLAB code to analyze P-V data sets by deriving an average P-V loop to simplify the representation of hemodynamic states over a wide range of time. This mathematical framework is informative and enables beneficial accessibility to P-V data analysis. However, an inherent limitation of this method is the forced structure of matrices such that all the P-V loop data are stored into an arbitrary or predefined size. This process often eliminates a considerable amount of data, which could potentially lead to erroneous data analyses and/or interpretations. We believe this method may be non-ideal when applied to longitudinal data from acute conditions such as hemorrhage and aortic occlusion studies. In these experiments, it is not uncommon to observe P-V loops that change in both size and number of data points. These changes are often correlated to periods of time when the subject is experiencing arrhythmias, bradycardia, or tachycardia, and thus having the ability to quantitatively assess these events is important.

Toward this goal, we developed a novel Python algorithm to quantify both the load-dependent and independent indices of cardiac function during hemorrhage and aortic occlusion. Our objective was to evaluate the variability in single-beat and longitudinal P-V loops during hemorrhagic shock, complete aortic occlusion with REBOA, and partial aortic occlusion with EVAC. To do so, we leveraged on a previously published porcine hemorrhage model (16). We hypothesized that the variability in P-V loop parameters would be greatest in animals receiving REBOA and that variability in cardiac metrics would be associated with poorer animal outcomes.

## Materials and methods

### Animal model

Yorkshire-cross swine (*n* = 18, 12 males and six females) (*Sus scrofa*; S & S Farms, Ramona, CA, USA) were used in this study. All animal experiments were conducted under the approval of the Institutional Animal Care and Use Committee at David Grant USAF Medical Center, Travis Air Force Base, California, and the animal care and use was in strict compliance with the Guide for the Care and Use of Laboratory Animals. All animals were acclimated for a minimum of 7 days prior to use; at the time of experimentation, the animals weighed 70–92 kg and were 4–6 months of age.

**Figure 1:**
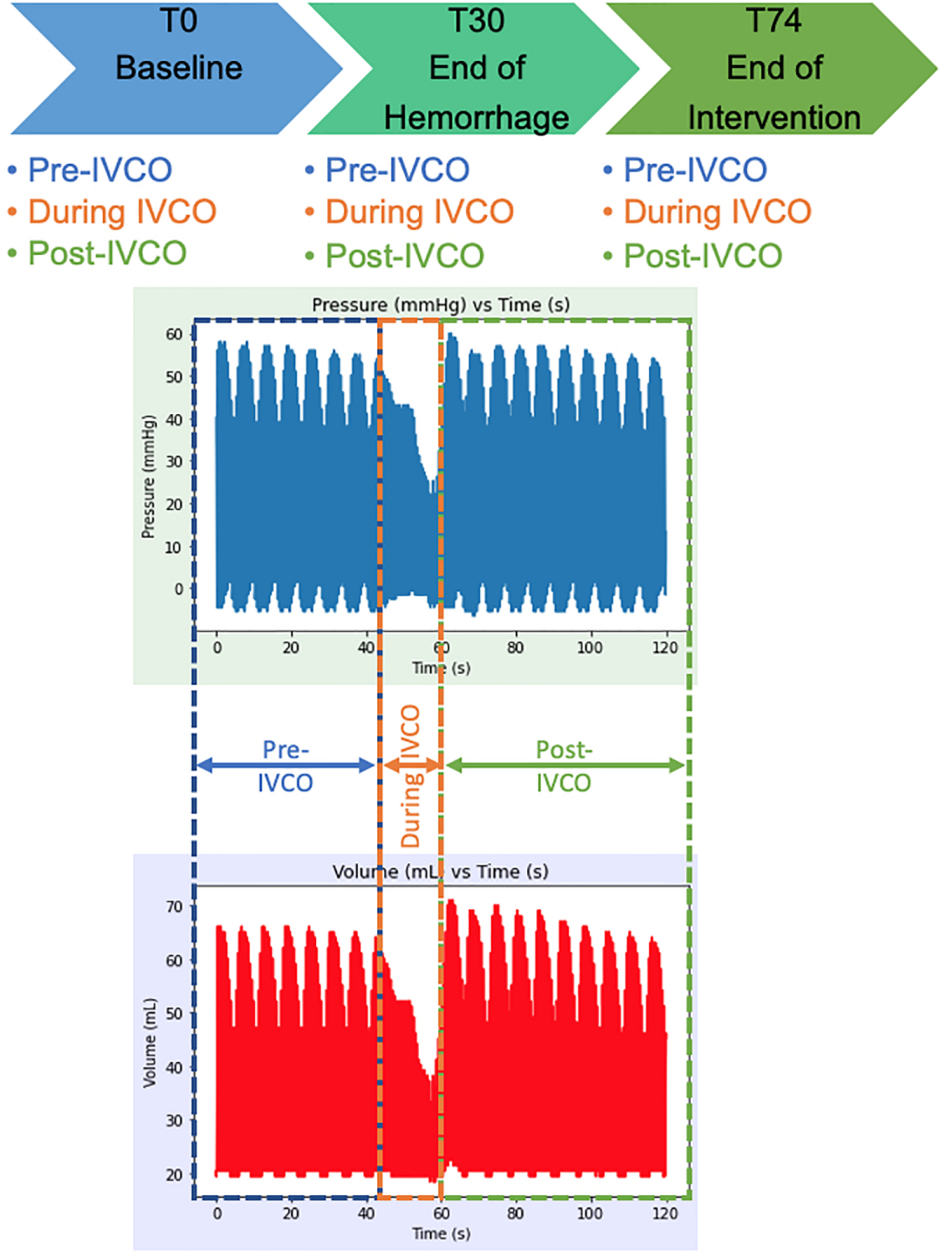
Experimental timeline and raw P-V loop data extraction time points. The P-V data was extracted from baseline (T0), at the end of a 25% hemorrhage (i.e., 30 min after baseline, T30), and after 44 min of intervention with REBOA or EVAC (i.e., T74). During each experimental time point, the data were extracted from the pre-IVCO, during IVCO, and post-IVCO. The average time of IVCO was 15 s. For pre- and post-IVCO segments, the data were sampled over 1 min.

The animals underwent controlled hemorrhage of a 25% blood volume (estimated at 60 ml/kg) over 30 min. During this period, the swine were randomized to receive one of three different treatment strategies for 45 min following hemorrhage: Zone 1 complete occlusion (REBOA, *n* = 6), Zone 1 automated partial occlusion (EVAC, *n* = 6), or no intervention (Control, *n* = 6). In each group, the sex breakdown was four males and two females. After intervention, the animals were resuscitated with shed blood, and the aortic balloons were gradually deflated over 5 min. Subsequently, all animals entered the critical care phase of the experiment where an automated critical care platform initiated isotonic fluid boluses and titrated vasopressors based on the same predefined algorithm. This phase lasted until the animal died or was euthanized 6 h after the start of the experiment. For the purpose of this study, we focused our analysis to the following three key time points: T0—baseline, T30—end of a 25% hemorrhage, and T74—end of intervention, as illustrated in Figure 1.

### Pressure–volume data acquisition

A P-V loop catheter (Transonic Systems Inc., Ithaca, NY, USA) was positioned in the left ventricle of the heart under fluoroscopic guidance, as previously described (16). The catheter was positioned in the long axis of the left ventricle with the tip at the apex (22). Consistent with the manufacturer's protocol, all catheters were hydrated in saline for at least 20 min prior to experimentation and were zeroed using the pressure balance controls in the ADV500 pressure–volume measurement system (Transonic). Blood resistivity was measured at the beginning of the experiment using the calibration probe connected to the ADV500 system. To measure the preload-independent metrics of cardiac performance, the right-sided cardiac inflow was occluded with the prepositioned inferior vena cava occlusion (IVCO) balloon during a ventilatory pause at predetermined time points. Data were acquired using the ADV500 pressure–volume measurement system (Transonic Systems Inc., Ithaca, NY, USA) and LabChart (ADInstruments, Colorado Springs, CO, USA). In addition to the P-V loop data, the heart rate, arterial pressures proximal and distal to the aortic balloon, central venous pressure, core temperature, and aortic flow were measured in real-time for the duration of the experiment. The full details describing the animal preparation, intervention, and critical care algorithm have been previously described by Beyer et al. (16).

### P-V loop data analysis

Our group developed a novel algorithm in Python capable of isolating, storing, and analyzing raw P-V loop data of variable sizes such that we could quantify the time-dependent changes. A 1 min of raw P-V data during the pre- and post-IVCO periods at each experimental time point was exported from LabChart into an .xlsx file (Microsoft Excel). An average of 15 s of data during the IVCO period was extracted as well. The IVCO period was generally brief during the experiment, and hence resulted in the shorter temporal files. All raw P-V data were sampled at 10 ms time steps, which provided adequate temporal resolution. The .xlsx files were then imported into Python using a Pandas data frame and converted into NumPy arrays to conduct our P-V loop analyses.

### Semi-automated and user-guided P-V loop data cleaning algorithm

Our mathematical framework converts the raw P-V loop data into polar coordinates, similar to Stonko et al. (21), allowing us to align each P-V loop data point to correspond to a singular point on a circular shape. The data are then analyzed within quadrants, where Quadrant IV refers to the start of the isovolumetric contraction and Quadrant I is the end of the isovolumetric contraction (see Supplementary Figures S1A,B). By extracting a single loop in this manner, we are able to define the end-systolic and end-diastolic ordered pairs and their respective time points.

We defined the end-systole and end-diastole using the minimum and maximum pressure gradient over time (i.e., dP/dt min and dP/dt max), similar to Abel's definition relying on the work of Gleason and Braunwald (23, 24). In the event that dP/dt did not render accurate end-systolic points, such as in cases where arrhythmic cardiac behavior was observed, we used the maximal elastance point (*E*_max_) method, developed by Suga and Sagawa (25) to define end-systole. Once end-systole and end-diastole were defined, the P-V loops were recreated using these starting and stopping time points. With this change in orientation, our algorithm was able to capture each cardiac cycle and give us beat-to-beat analytics.

The algorithm is designed to prompt the user if P-V loops appeared aberrant or irregular, which allowed for a level of quality check. The settings for this user-guided check could be set that a user could preview each P-V loop prior to ensemble averaging and performing the analytics (see Supplementary Figure S2). Once the quality of P-V loops was verified, we quantified the following cardiac parameters from each P-V loop: CO, ejection fraction (EF), arterial elastance (Ea), stroke volume (SV), and stroke work (SW).

### Ensemble-averaged P-V loops

#### For each animal (within-subject variability)

An ensemble averaging algorithm was developed to best illustrate the behavior of P-V loops sampled for each pig and experimental time point. This ensemble average is defined by calculating the mean array size of the P-V loop data and then generating the ensemble-averaged P-V loop with the average array size. The algorithm is designed such that it can reshape the number of data points from each P-V loop, while preserving the P-V shape and allowing for consistent averaging. This representation helps account for P-V loop variability (where we get both the mean and standard deviation) for each pig at each time point thus, creating a singular representation of the P-V loop for a specific phase. A sample ensemble-averaged P-V loop in comparison with its originating raw data can be found in Supplementary Figure S3.

#### For each intervention group (within-group variability)

Once the individual ensemble averages were created for each pig, a second ensemble averaging algorithm was conducted to get a representative average behavior of P-V loops within each intervention group. Here, the P-V loops from all six pigs in each group were averaged at each experimental time point. This algorithm works similar to the aforementioned algorithm and renders the mean and standard deviation P-V relation for each group.

### Quantifying load-independent cardiac metrics

The end-systolic pressure–volume relationship (ESPVR) and end-diastolic pressure–volume relationship (EDPVR) are the two key load-independent cardiac performance metrics. Given that ESPVR and EDPVR are load-independent cardiac metrics, we analyzed these during IVCO periods only. Our algorithm was optimized to simultaneously plot several models for both ESPVR and EDPVR (see Supplementary Table S1). From these options, we reported ESPVR through a linear regression to represent it in the form:

(1) $$P_{es}=E_{es}(V_{es}+V_{0})$$

where *E_es_* is the slope of this line, referred to as the left ventricular elastance at end-systole. *V_es_* is the end-systolic volume (ESV), *P_es_* is the end-systolic pressure, and *V*_0_ is the volume at zero pressure, which is commonly referred to as the volume-axis intercept. Given that we exposed our animals to hemorrhagic shock, we used the data at the “end of hemorrhage” (i.e., T30) to best identify *V*_0_. Once *V*_0_ was determined for each animal, we constrained the remaining linear fits with that unique *V*_0_ such that we preserved the physiologic relevance of the ESPVR linear fits.

EDPVR was calculated using a two-parameter cubic regression to fit the form:

(2) $$P_{ed}=D+aV_{ed}^{3}$$

where *V_ed_* is the end-diastolic volume in the left ventricle, *D* is the pressure-axis intercept, *a* is a constant, and *P_ed_* is the end-diastolic pressure.

### Statistical analysis

The cardiac performance metrics are summarized using mean and standard deviations (i.e., mean ± SD). Box plots were used to illustrate the range and variability of each measured or calculated cardiac metric. We ran ANOVA and reported the *F*-test for comparison of variances of cardiac metrics between experimental groups. To estimate the effects of REBOA and EVAC interventions on the changing cardiac function of the pig over time, we ran a mixed linear model containing a group-time interaction term. If the group-time interaction was not statistically significant, we then performed a mixed linear model without the interaction term to assess the main effects of the intervention group and time. Both models were adjusted for the sex and weight of the animal, and an autoregressive correlation matrix was employed to account for repeated measures. The selection of an autoregressive correlation matrix was deemed to be most appropriate for analyzing the repeated measures and time series data in this study. The control group was set as the referent group, and T0 (i.e., baseline) was set as the referent time point. Variables were either square root or log transformed to meet the linear regression assumptions of normality.

To determine if there were any differences in cardiac function parameters between pre-, during-, and post-IVCO periods, we performed a similar mixed linear regression model. Here, the mixed linear regression model was stratified by the intervention group and adjusted for sex and weight. Similar to before, we tested for IVCO–time interactions, and if they were not significant, then the main effects for IVCO period and time were reported without the interaction term. A summary of hemodynamic parameters across all groups at all time points can be found in Supplementary Tables S2A–C.

Lastly, to determine whether any of the cardiac metrics at the end of intervention (i.e., T74) (and their variability) were associated with resuscitation needs at the end of study, we performed pairwise correlations. The statistical significance was set at *p* < 0.05 level for all regression models. All graphs were made in Python using Matplotlib (26) with the Seaborn package (27). The statistical analysis was performed using STATA (version 17.0; College Station, TX, USA).

## Results

**Figure 2:**
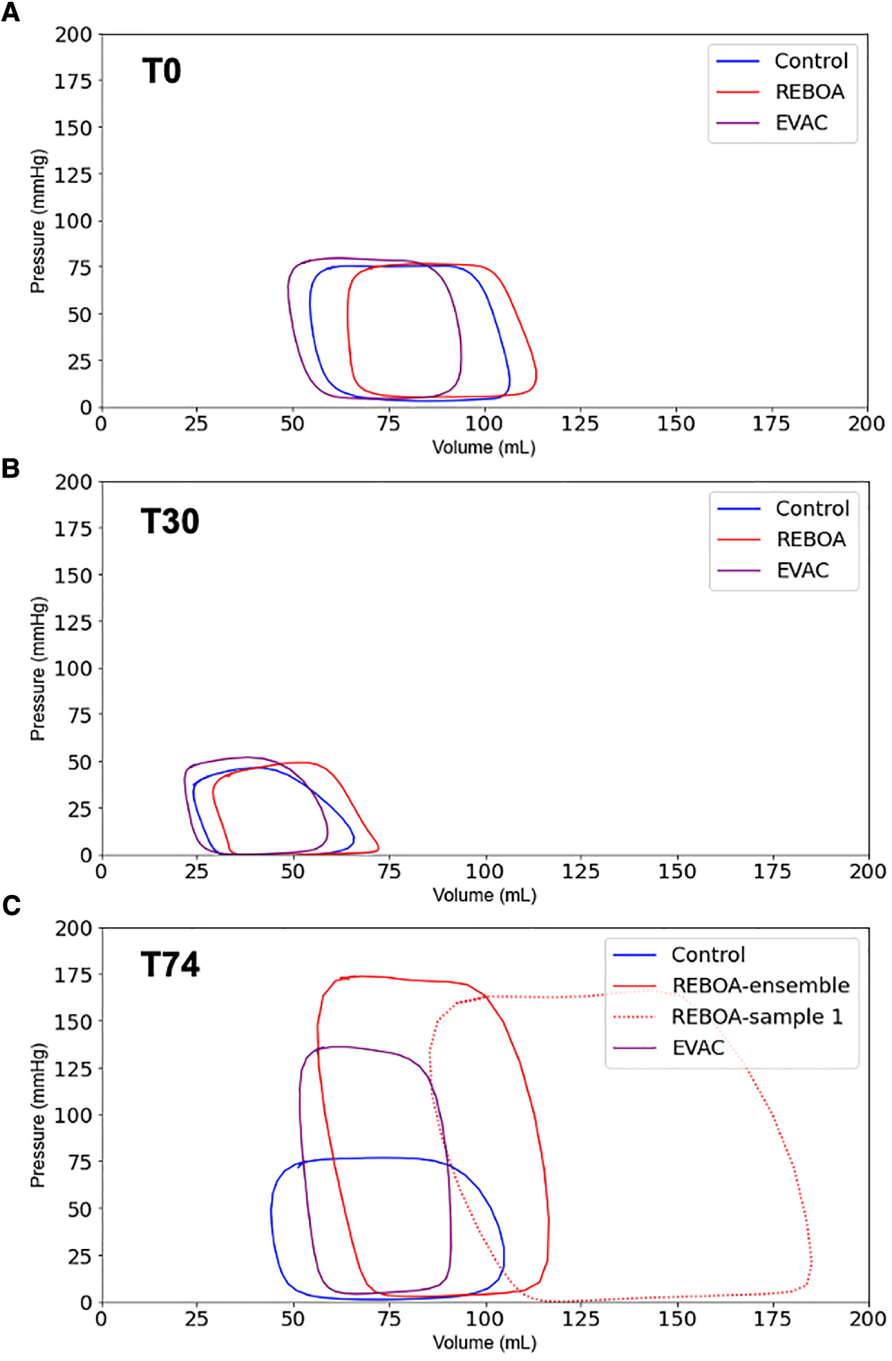
Ensemble average P-V loops. The pressure–volume loops were averaged for all pigs in the three intervention groups: Control (blue), REBOA (red), and EVAC (purple) (*n* = 6/group). These figures illustrate the ensemble-averaged P-V loops at (**A**) baseline (T0), (**B**) end of hemorrhage (T30), and (**C**) end of intervention (T74) for each group. As expected, the P-V loops get smaller at the end of hemorrhage, reflective of lower pressures and volumes, followed by a significant increase in pressure and volume with the REBOA intervention. (**C**) illustrates the averaged P-V loops in each group as well as one of the REBOA pigs (red-dotted line), which illustrates a major deviation from the “averaged” P-V response. A significant amount of subject variability was observed in the REBOA group.

The ensemble average P-V loops for each intervention group at baseline, end of hemorrhage, and end of intervention are calculated and illustrated in Figure 2. At baseline, we observed similarly shaped P-V loops; however, there was a variability between groups in the left ventricular volumes (Figure 2A). While the baseline end-systolic pressures (ESPs) were comparable between the control, REBOA, and EVAC groups, the left ventricular volumes ranged from 50 to 110 ml. We found that some of this variability was significantly associated with the body weight of the animals (*p* < 0.05). As expected, by the end of a 25% hemorrhage at T30, all animals illustrated a major leftward and downward shift in their left ventricle P-V loop (Figure 2B). The left ventricular volumes ranged from 25 to 75 ml, and the ESPs ranged from 30 to 50 mmHg. At the end of each intervention (i.e., T74), the ensemble P-V loops are corrected for the left ventricular volume to resemble near-baseline characteristics. However, the peak pressures are significantly elevated, as shown in Figure 2C. In the control group, the P-V loops at T74 are wider compared with T0. In the REBOA group, P-V loops at T74 exhibited significantly higher maximum pressure values (up to 175 mmHg), making them at least two times taller compared with T0. However, this effect was highly variable, as shown in Figure 2C, where one animal in the REBOA group (dotted red line) displayed major increases in both left ventricular pressure and volume, illustrating within-group and between-subject variability. This illustrates the importance of evaluating both individual and group variability within this hemorrhage and aortic occlusion animal model. Finally, the pigs in the EVAC group experienced a similar increase in the peak end-systolic pressure at T74, albeit with less variability and a tighter range of the left ventricular volumes.

### Variations in the end-systolic and end-diastolic pressures and volumes by the intervention group

**Figure 3:**
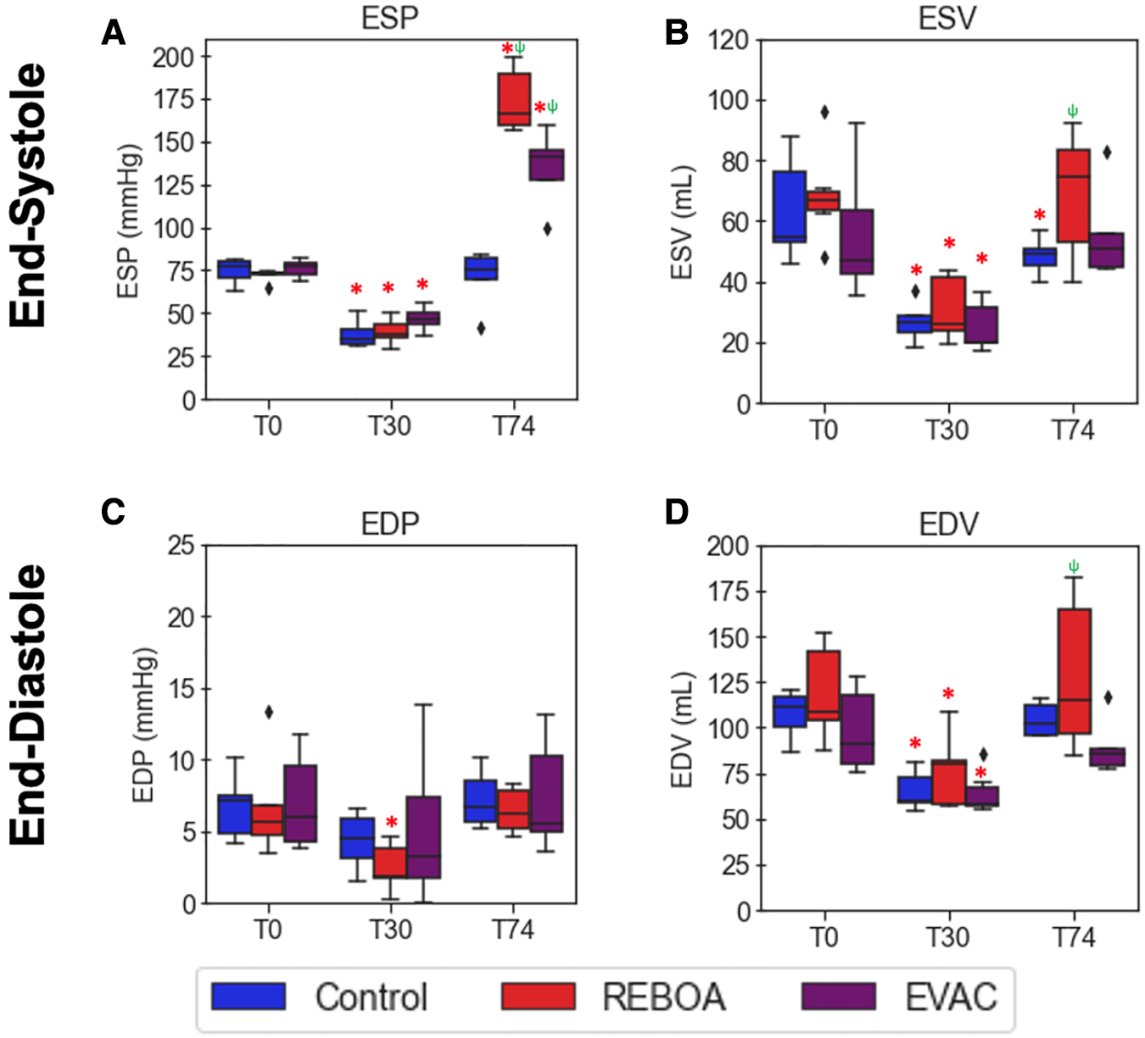
Comparison of end-systolic and end-diastolic pressures and volumes by intervention group. Average end-systolic (**A**) pressure (ESP) and (**B**) volume (ESV) as well as end-diastolic (**C**) pressure (EDP) and (**D**) volume (EDV) are plotted over time by group (*n* = 6/group). The red asterisks (*) represent statistically significant differences between time, where T0 (baseline) is the referent time. The significant effects between groups are illustrated with a green psi (ψ) symbol, where the control group is the referent group. The diamond-shaped crosses are extreme data points from each group. (**A**) After hemorrhage in all treatment groups, ESP significantly decreased by an average of 50.7 mmHg with a 95% CI of −58.4 to −41.7 mmHg (*p* < 0.001). At T74, the REBOA group experienced an average increase in ESP by 140.2 mmHg (95% CI: 102.7–184.6 mmHg, *p* < 0.001), while EVAC was associated with an average increase of 76.4 mmHg (95% CI: 47.5–110.9 mmHg, *p* < 0.001). (**B**) ESV was found to have significantly decreased after hemorrhage in all groups, with an average decrease of 57.7 ml (95% CI −63.7 to −50.7 ml, *p* < 0.001). In all groups at T74, however, there were no significant differences with the baseline conditions. (**C**) There were no significant differences in EDP when compared with baseline conditions in all groups, with the exception of the REBOA group at T30; however, the average across all groups at T30 was not significantly decreased when compared with the baseline state, with a pooled decrease of 2.35 mmHg (95% CI −4.8–13 mmHg, *p* = 0.064). (**D**) EDV was found to have significantly decreased after hemorrhage in all groups, with an average decrease of 42.7 ml (95% CI −56.0 to −29.7 ml, *p* < 0.001). In all groups at T74, however, there were no significant differences with the baseline conditions.

Focusing on the 1 min of pre-IVCO data at each respective experimental time point, we evaluated the variations in the end-systolic and end-diastolic conditions within each group. The ESP values were comparable between groups at both baseline and the end of hemorrhage (Figure 3A). As expected, ESP significantly decreased over time after hemorrhage in all groups by on average 50.7 mmHg with a 95% confidence interval (CI) of−58.4 to−41.7 mmHg, *p* < 0.001. The intervention groups REBOA and EVAC at T74 resulted in a significant increase in ESP compared with the baseline values. REBOA at T74 was associated with an average increase of 140.2 mmHg in ESP (95% CI: 102.7–184.6 mmHg, *p* < 0.001), while EVAC was associated with an average increase of 76.4 mmHg in ESP (95% CI: 47.5–110.9 mmHg, *p* < 0.001). When compared with the control group at T74, REBOA was associated with an average increase of 144.9 mmHg in ESP (95% CI: (106.5–190.3 mmHg, *p* < 0.001), while EVAC was associated with an average increase of 97.6 mmHg in ESP (95% CI: 64.6–137.2 mmHg, *p* < 0.001). At T74, the REBOA group displayed not only the highest average ESP but also the greatest within-group variability (173.8 ± 18.9 mmHg, *F* = 171.45 *p* < 0.0001) followed by the EVAC group (134.6 ± 22.4 mmHg, *F *= 65.98, *p* < 0.0001). The control group had the least variability (71.6 ± 15.8 mmHg).

Unlike ESP, the ESV levels were more variable both between groups and over time (Figure 3B). Substantial variability in ESV was observed at baseline in the control and EVAC groups, which subsided after hemorrhage. ESV was found to have significantly decreased after hemorrhage in all groups [on average, decrease by −57.7 ml with a 95% CI of −63.7 to −50.7 ml, *p* < 0.001. However, following the REBOA intervention at T74, we observed a significant increase in EDVs, compared with T30, with notably variability over time. When compared with the control group at T74, REBOA was associated with an average increase of 20.6 ml in ESV (95% CI: 2.5–38.8 ml, *p* = 0.026), while EVAC was only associated with an average increase of 8.5 ml in ESV (95% CI: −10.8 to 27.7, *p* = 0.389). On average, the highest ESV was observed in the REBOA group (68.9 ± 21.1 ml) at T74.

On average, the end-diastolic pressure (EDP) in the control group (7.2 ± 1.9 mmHg) was similar to that of the REBOA group (6.4 ± 1.6 mmHg) and EVAC group (7.5 ± 4.0 mmHg) at T74 (end of intervention). Interestingly, EDP was not significantly different between groups or across time (all *p* > 0.05). This may be partly due to the variability seen in EDP and EDVs throughout the groups and experimental time points. On average, at T74, the highest EDV was observed in the REBOA group (128.3 ± 43.3 ml), followed by the control group (104.3 ± 9.3 ml). The EVAC group (89.8 ± 15.7 ml) had the lowest EDV and the least group variance at T30 and T74.

### REBOA is associated with greater variation in cardiac function metrics

**Figure 4:**
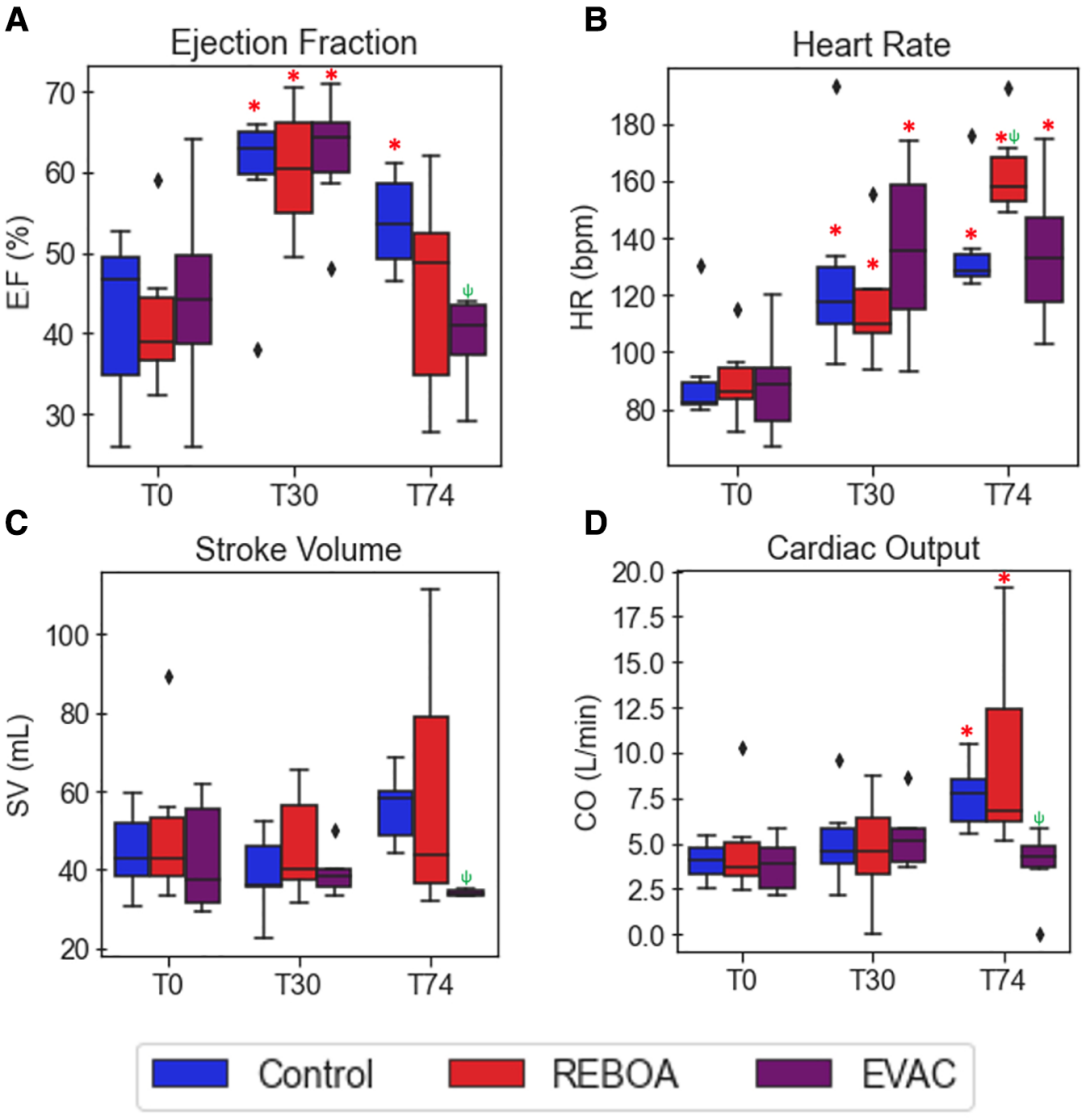
Summary of cardiac performance metrics by intervention group over time. Box plots of (**A**) EF, (**B**) HR, (**C**) SV, and (**D**) CO at pre-IVCO time periods are illustrated over time and across groups. The red asterisks (*) represent statistically significant differences between time, where T0 (baseline) is the referent time. The significant effects between groups are illustrated with a green psi (ψ) symbol, where the control group is the referent group. The diamond-shaped crosses are extreme data points from each group. (**A**) EF was found to significantly increase after hemorrhage in all groups by an average of 16.8% with 95% CI of 8.0%–25.6% (*p* < 0.001). At T74, while EF did decrease when compared with the hemorrhage state, it was still significantly higher than that observed in the baseline state by 11.6% with 95% CI of 2.8%–20.4% (*p* = 0.01). The REBOA and EVAC groups, on the other hand, did not lead to a significant decrease in EF at T74 compared with the baseline conditions for both groups (*p* = 0.460 and *p* = 0.206, respectively). (**B**) HR was found to have significantly increased after hemorrhage by an average of 38.4 bpm with 95% CI of 19.7–60.1 bpm (*p* < 0.001). At T74, HR was elevated in in all the three intervention groups. The HR in the REBOA group increased by an average of 83.2 bpm in HR (95% CI: 58.5–111.9 bpm, *p* < 0.001). The EVAC group was associated with an average increase of 55.4 bpm in HR (95% CI: 33.1–81.5 bpm, *p* < 0.001) compared with baseline. By comparison, the control group experienced an increase of 50.8 bpm in HR (95% CI: 30.4–74.4 bpm, *p* < 0.001) compared with baseline. (**C–D**) There were no significant differences in SV (**C**) and CO (**D**) when compared with baseline across the three intervention groups.

In Figure 4, the variations in load-dependent cardiac performance metrics are explored. Beginning with the EF, we observed considerable variability across time and between groups (Figure 4A). EF significantly increased by 16.8% after hemorrhage in all groups (95% CI: 8.0%–25.6%, *p* < 0.001). At T74, the EVAC group had significantly lower EF compared with the control group (*p *= 0.004). The use of REBOA, on the other hand, did not lead to a significant decrease in EF at T74 compared with the control group (*p* = 0.168). We attribute this non-statistically significant finding to the high level of within-group variability in EF within the REBOA group (45.2% ± 13.4% at T74).

The heart rate variability was relatively low at baseline but increased over time. Expectedly, the 25% hemorrhage resulted in a significant increase in HR across all groups (38.4 bpm with 95% CI: 19.7–60.1 bpm, *p* < 0.001). At T74, HR remained elevated in all the three intervention groups. The REBOA group at T74 was associated with an average increase of 83.2 bpm in HR (95% CI: 58.5–111.9 bpm, *p* < 0.001), while EVAC was associated with an average increase of 55.4 bpm in HR (95% CI: (33.1–81.5 bpm, *p* < 0.001) compared with baseline. Contrary to previous findings, the EVAC group presented with the most HR variability at T30 and T74, as illustrated in Figure 4B.

There were no significant differences in SV or CO at baseline across the three intervention groups. The pooled average baseline SV was 45.9 ± 15.3 ml, and the average CO was 4.2 ± 1.9 L/min. Variability in SV and CO were comparable between groups at both T0 and T30 (*F* = 2.59, *p* = 0.09 for SV; and *F* = 2.83, *p *= 0.075 for CO at T0). However, the REBOA group presented with significant increases in the variance of SV and CO at T74 (Figures 4C,D). At T74, SV was 59.4 ± 33.2 ml, and CO was 9.7 ± 5.6 L/min in the REBOA group contrastingly to the EVAC group that had very little within-group variability and the smallest SV and CO at T74. Importantly, the weight of the animal was significantly associated with SV and CO, where each kilogram increase in body weight was associated with an average increase of 2.5 ml in SV (95% CI: 0.5–4.7 ml, *p* = 0.016) and 237.9 ml/min increase in CO (95% CI: 51.4–424.4 ml, *p* = 0.012).

**Figure 5:**
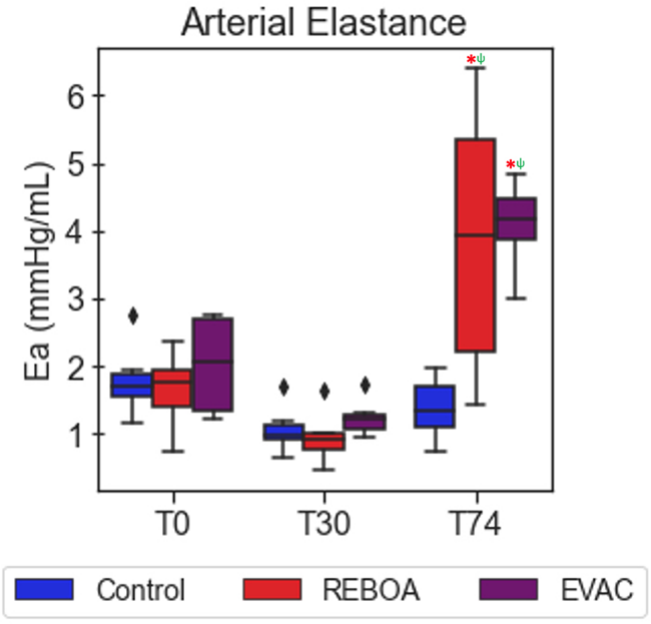
Arterial elastance varies significantly after REBOA intervention. The average arterial elastance was calculated as the ratio of the end-systolic pressure to stroke volume. As expected, arterial elastance is comparable between pigs in all the three intervention groups at baseline (T0) and at the end of a 25% hemorrhage (T30). However, the introduction of REBOA and EVAC significantly increased the calculated arterial elastance to levels nearly three times higher than baseline. Data presented are from the pre-IVCO period. The red asterisks (*) represent statistically significant differences between time, where T0 (baseline) is the referent time. The significant effects between groups are illustrated with a green psi (ψ) symbol, where the control group is the referent group, and the diamond-shaped crosses are extreme data points from each group.

In Figure 5, the variations in Ea were explored between intervention groups and over time. Significant increases in Ea were observed in the REBOA and EVAC groups, compared with the baseline values. REBOA at T74 was associated with an average increase of 2.2 mmHg/ml (95% CI: 1.4–3.1, *p* < 0.001), while EVAC was associated with an average increase of 2.1 in Ea (95% CI: 1.2–3.1, *p* < 0.001). When compared with the control group at T74, REBOA was associated with an average increase of 2.4 mmHg/ml in Ea (95% CI: 1.5–3.3, *p* < 0.001), while EVAC was associated with an average increase of 3.02 mmHg/ml in Ea (95% CI: 2.1–4.0, *p* < 0.001). Notably, the REBOA group exhibited the greatest variation in Ea at T74, potentially due to the considerable within-group variability in both the end-systolic pressures and stroke volumes following REBOA usage. Furthermore, the body weight of the animal was significantly associated with lower Ea of −0.064 mmHg/ml (95% CI: −0.1 to −0.02, *p* = 0.005), regardless of intervention group.

SW was found to have significantly decreased after hemorrhage in all groups (approximately 1,849.3 mmHg ml with 95% CI: −3,095.3 to −603.3 mmHg ml, *p* = 0.004). REBOA at T74 was associated with an average increase of 5,680.0 mmHg/ml with 95% CI of 4,434.0–6,926.0 mmHg ml (*p* = 0.034), while EVAC was associated with an average increase of 1,423.1 mmHg ml with 95% CI of 107.9–2,738.3 mmHg ml (*p* = 0.034). Compared with the control group at T74, REBOA was associated with an average increase of 5,268.5 mmHg ml with 95% CI of 3,953.6–6,583.4 mmHg ml (*p* < 0.001), while EVAC was associated with an insignificant average increase of 8.3 mmHg ml with 95% CI of −1,403.0 to 1,419.5 mmHg ml (*p* = 0.991). At T74, on average, SW was found to be highest with REBOA (9,327.8 ± 3,237.8 mmHg ml) followed by EVAC (4,696.4 ± 825.3 mmHg ml) and then the control group (4,771.3 ± 870.8 mmHg ml). Furthermore, weight had an impact on the SW in all groups of 111.4 mmHg ml (95% CI: 43.4–179.4 mmHg ml, *p* = 0.001).

### Comparison of cardiac parameters pre-IVCO and post-IVCO

**Figure 6:**
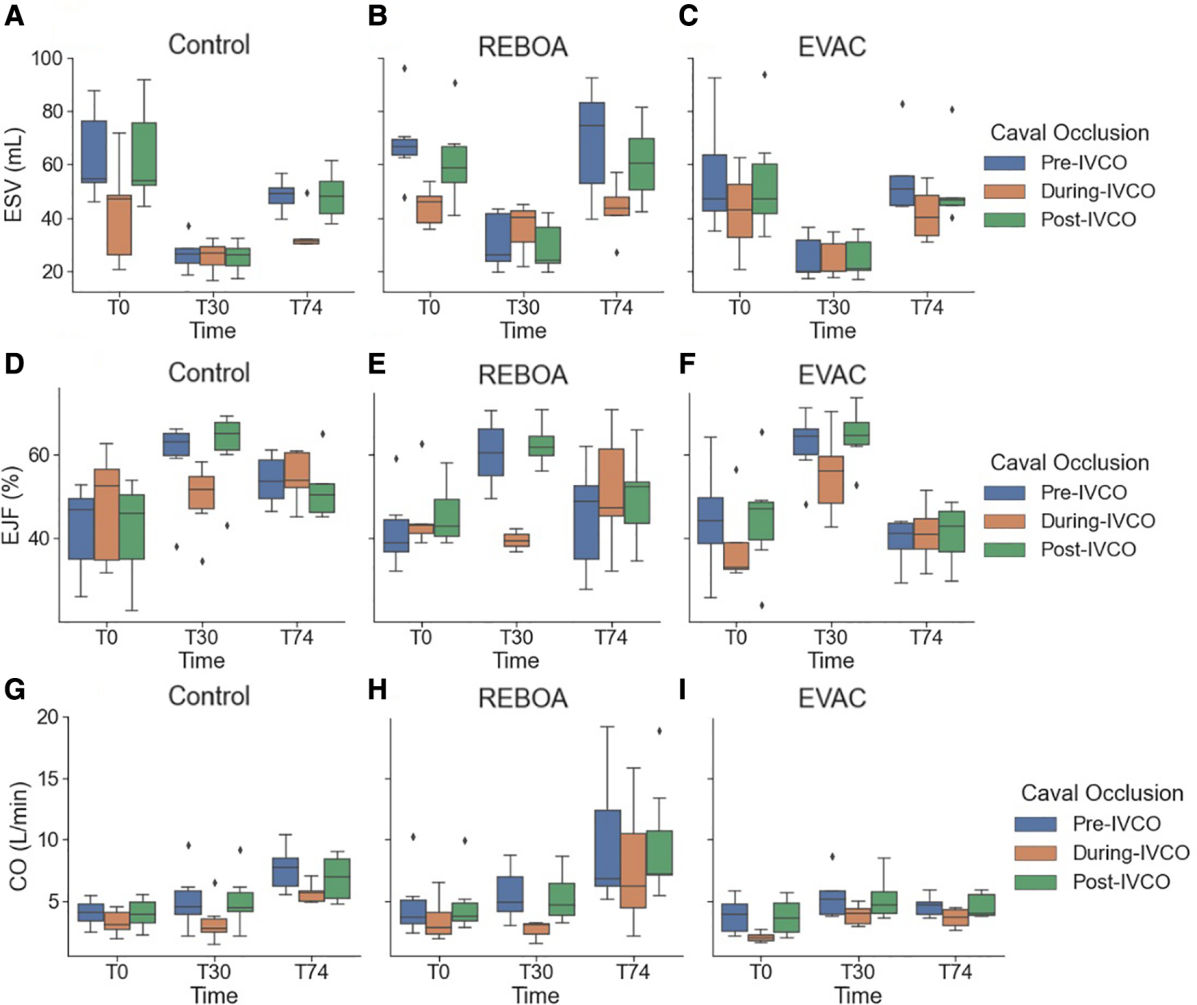
A summary of the key cardiac performance metrics pre-IVCO, during IVCO, and post-IVCO. We summarize the key cardiac parameters: (**A–C**) ESV, (**D–F**) SV, and (**G–I**) CO for each intervention group during the pre-IVCO (blue), during IVCO (orange), and post-IVCO (green) periods. As illustrated, we observed a high degree of variation in ESV at baseline. The stroke volume was most variable at T74 across all the intervention groups. We did not observe any statistically significant differences in ESV, SV, or CO values between pre-IVCO and post-IVCO periods.

Figure 6 features three key cardiac performance metrics, namely, ESV, EF, and CO, which demonstrate the absence of statistically significant differences pre- and post-IVCO. In the brief implementation of IVCO, it was found that there was no significant effect on any of the hemodynamic parameters when comparing pre- against post-IVCO. While we did observe some trends where the cardiac metrics are generally lower in magnitude under IVCO conditions, they were not statistically different from non-IVCO periods.

When comparing pre-IVCO measurement of parameters against those observed during IVCO, certain parameters demonstrated no changes. EDP was found to have decreased significantly during IVCO implementation at BL, with an average decrease in 17.4 mmHg with 95% CI of −27.1 to −7.7 (*p* = 0.002), while during hemorrhage and T74 in all groups, there was an insignificant decrease. ESP, on the other hand, was found to have decreased significantly during IVCO implementation in all groups at all experimental time points (*p* < 0.05).

Conversely, ESV showed significant decreases during IVCO implementation, with a significant average decrease of 17.4 ml (95% CI: −27.1 to −7.7 ml, *p* = 0.002) during baseline and by 5.2 ml (95% CI: −9.5 to −0.9 ml, *p* = 0.022) during hemorrhage. Similar trends were found in the control and REBOA groups at T74, but in the EVAC group, a mild increase of 7.4 ml (95% CI: −12.2 to 26.9 ml, *p* = 0.415) was observed (which was not statistically significant), implying that during IVCO end-systolic volumes in EVAC treatment groups were relatively constant. This unique nature of the EVAC group was also observed in the EDV, where an insignificant decrease of 1.4 ml (95% CI: −23.5 to 20.7 ml, *p *= 0.89) was observed, whereas the other groups had significant decreases in EDV. To visualize the P-V loop differences pre-IVCO, during IVCO and post-IVCO, we refer the reader to Supplementary Figure 3.

### REBOA is associated with the highest ESPVR slope and variations in LV contractility

The ESPVR was quantified using a two-parameter linear regression, as shown in Equation 1. The average and standard deviations of the parameters in each group are reported in Table 1. In a linear ESPVR, *E_es_* is customarily used as an index with a direct relationship with the left ventricular contractility (inotropy), a key factor that impacts myocardial performance. Positive increases in *E_es_* are indicative of increases in the slope of the ESPVR and hence a growth in contractility; conversely, decreases in this term are indicative of decreases in contractility. *V*_0_ represents the volume-axis intercept (x-intercept), as opposed to the pressure-axis intercept (y-intercept). *V*_0_ terms were identified for each subject at T30 and then constrained during the other time points (i.e., T0 and T74) to maintain physiologic relevance and allow for comparison across groups.

**Table 1:** A summary of curve fit parameters for the calculation of ESPVR during IVCO.

Time point	Group	ESPVR		Comparison between groups	Comparison over time
		$P_{es}=E_{es}(V_{es}+V_{0})$			
		*E*_es_ (mmHg/ml)	*V*_0_ (ml)	*p*	*p*
T0 baseline	Control	1.70 ± 0.70	13.30 ± 2.81	—	—
	REBOA	1.44 ± 0.56	21.27 ± 7.49	0.747	—
	EVAC	2.32 ± 0.13	18.86 ± 6.00	0.687	—
T30 end of hemorrhage	Control	3.47 ± 0.22	13.30 ± 2.81	—	0.009
	REBOA	2.78 ± 1.18	21.27 ± 7.49	0.882	0.013
	EVAC	4.32 ± 0.89	18.86 ± 6.00	<0.0001	<0.0001
T74 end of intervention	Control	1.90 ± 0.69	13.30 ± 2.81	—	0.470
	REBOA	3.28 ± 0.07	21.27 ± 7.49	0.241	0.018
	EVAC	3.91 ± 1.25	18.86 ± 6.00	0.003	<0.0001

The mean and standard deviations are reported for each parameter at baseline (T0), end of hemorrhage (T30), and at the end of intervention (T74), and for each experimental group. The control group was our referent group for comparisons of *E_es_* between groups, and T0 was our referent group for comparisons of *E_es_* over time.

**Figure 7:**
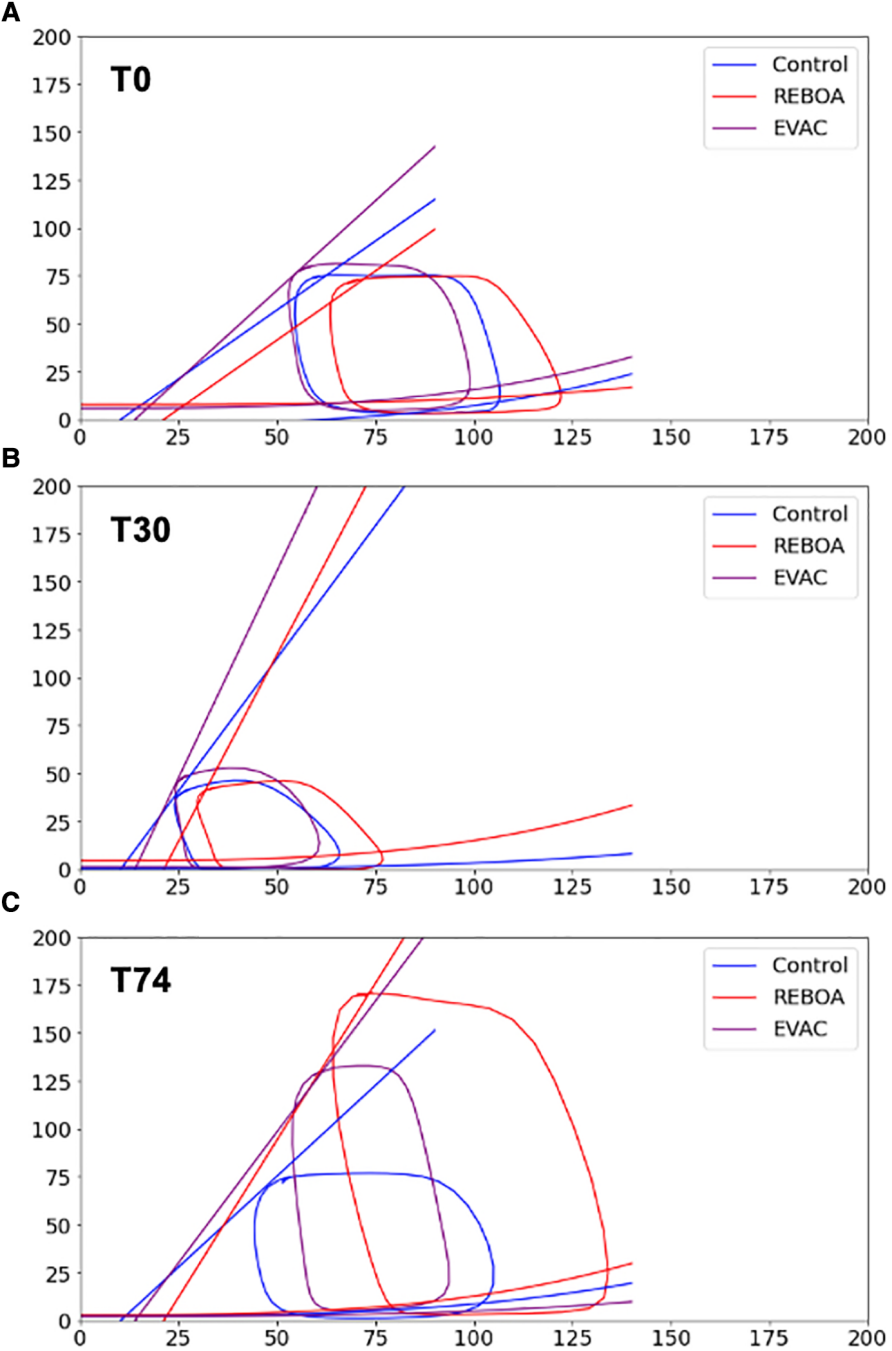
ESPVR and EDPVR by intervention group. The ESPVR and EDPVR for each group was applied to the ensemble averages at (**A**) baseline, (**B**) end of hemorrhage, and (**C**) end of intervention. For all cases, a linear ESPVR was fitted using the equation: $P_{es}=E_{es}(V_{es}+V_{0})$. EDPVR was fitted using a cubic equation: $P_{ed}=D+aV_{ed}^{3}$. As expected, the left ventricular elastance (i.e., *E_es_*, slope of ESPVR) increased after hemorrhage in all three groups, nearly doubling in value. After the intervention was performed (i.e., T74), the ESPVR slopes in the control group decreased to near-baseline levels but remained elevated in both the REBOA and EVAC groups, implying an increased cardiac contractility. No discernable differences between EDPVR were observed between groups or over time.

At baseline, the left ventricular elastance (*E_es_*) across all animals and groups were relatively the same. No significant differences were observed between groups. During hemorrhage, increases in *E_es_* were observed in all groups, with an average doubling in value from baseline to end of hemorrhage. This was to be expected since contractility increases during hemorrhage. Finally, at the end of the intervention (i.e., T74), there were different responses depending on the intervention group. In the control group, *E_es_* returned to near-baseline levels, whereas *E_es_* remained elevated in both REBOA and EVAC groups. In fact, the EVAC animals had significantly higher *E_es_* values compared with control animals at both T30 and T74 time points (Table 1). A visual illustration of the changes in slope (i.e., Ees) can be appreciated in Figure 7, where we have graphed the average linear ESPVR and cubic EDPVR fits for the ensemble average P-V loop from each intervention group and at each time point (Figure 7).

## Discussion

The left ventricular P-V loop data is the recognized standard for the direct and real-time measurement of cardiac function. P-V measurements can be made under steady state and IVCO settings, allowing one to determine the cardiac output and contractility of the heart under load-dependent and load-independent conditions. While this is the gold standard, the analysis of P-V loops and ESPVR are not trivial and can be particularly challenging in longitudinal studies where there can be significant variability both within subjects and across groups. In this study, we successfully developed a novel Python algorithm to quantify both the load-dependent and independent indices of cardiac function during baseline, hemorrhage, and aortic occlusion. We were able to quantify both individual variability using beat-to-beat analysis and group variability in longitudinal P-V loops during hemorrhagic shock, complete aortic occlusion with REBOA, and partial aortic occlusion with EVAC. We found that EF, ESV, and EDV were the most variable cardiac metrics between animals at baseline (or regardless of interventions). Consistent with our hypothesis, we found that animals receiving a complete occlusion with REBOA displayed greater variability in their cardiac function indices at T74, compared with the EVAC group. In general, the use of REBOA was associated with profound changes in the P-V relationship, often resulting in higher blood pressures than seen at baseline, or normal conditions.

In this study, we successfully evaluated the variability in P-V relationships within animals and across groups. At baseline, all animal groups should have been similar, and indeed our results confirm that the inherent physiologic variability in CO and P-V relationships is relatively low, which is on average the CO is 4.2 ± 1.9 L/min across all animals (*F* = 2.83, *p* = 0.075). We observed no significant differences in the cardiac function metrics between sex, but the body weight of the animal was associated with an average increase of 0.24 L/min in CO and a lower Ea of −0.064 mmHg/ml. In fact, we discovered that CO, SV, Ea, and SW were the four primary cardiac parameters that were significantly associated with the body weight of the animals, where for every 1 kg increase in body weight, we observed an average change in CO (increase of 0.24 L/min, *p* = 0.012), SV (increase of 1.5 ml, *p* = 0.006), Ea (−0.06 mmHg/ml, *p* = 0.005), and SW (111.4 mmHg ml, *p* = 0.001). The impact of body weight on these cardiac indices demonstrates an inherent physiologic variability that can be observed in all animal models including the Yorkshire swine. The inherent physiologic variability of each animal should be considered during experimental study designs and serve as a cautionary note to investigators performing research with animals. Potential confounding variables, such as bodyweight, age, and sex, should be controlled to the best of one's ability. Furthermore, sample sizes should be determined *a priori* based on robust power calculations. However, we found consistency in cardiac function measurements at baseline and end of hemorrhage across groups, which demonstrates the rigor of instrumentation and automated experimental methodology to enable a reproducible hemorrhagic shock model (28). While there was decreased variability for most parameters after the controlled hemorrhage in all groups, there were some notable exceptions such as HR. The elevated HR variability was not expected in a state of shock as others, such as Batchinsky et al. and Carrara et al. (29, 30), have shown that acute stress from hemorrhagic shock can impair the autonomic nervous system and its modulation of heart rate. The consistency in cardiac function measurements at baseline and end of hemorrhage also demonstrates the rigor of the instrumentation and automated experimental methodology to enable a reproducible hemorrhagic shock model (28, 30, 31). Collectively, the results from this suggest that an *n* = 6 pigs/group is only adequately powered to detect a twofold increase in cardiac performance metrics (e.g., SV, CO). In order to achieve 80% power at the 0.05 significance level, for smaller effect sizes (such as in the 1.2–1.5 range), we would recommend having an *n* = 15/group. The choice of the primary outcome and its associated variability is the key for future planning and study designs.

At the end of the 25% total blood volume hemorrhage, we observed significant decreases in ESP, ESV, EDP, EDV, and SW. This is consistent with a decrease in the preload and afterload conditions. Conversely, statistically significant increases in HR, EF, and *E_es_* were observed, with the REBOA group displaying the highest variability in these indices. As a result, the CO at the end of hemorrhage was not statistically different from that of the baseline values, indicating that the natural response of the animal is to preserve CO during acute hemorrhage. This is not surprising given that a 25% total blood volume hemorrhage falls under “compensatory shock” and is considered a Class II hemorrhage (15%–30% blood volume loss). The acute catecholaminergic response elevates HR and peripheral vasoconstriction to compensate for the volume loss (32–34). However, when subjected to a Class III or IV hemorrhage (30%–40% or >40% blood volume loss), these intrinsic mechanisms are no longer capable of compensating for the blood loss, and the animals subsequently experience “decompensatory shock” and a significant reduction in CO (35–37). It should be noted that within this study, all animals experienced an acute “shock” state, with a reduction in mean arterial pressure (MAP <40 mmHg), in conjunction with an elevated HR and lactate levels (>170 bpm and 3–4 mmol/L, respectively), similar to our prior published work (16).

The largest differences in cardiac function between groups were observed following the intervention phase (i.e., T74 min). In the control animals, the ESP, EDV, and EDP were restored to near-baseline levels, but CO was generally higher than baseline. Furthermore, animals in the REBOA group required significantly more resuscitation fluid when compared with the control and EVAC groups, as previously reported in Beyer et al. (16). This need could possibly be related to the high variability in cardiac performance seen in the REBOA group. Importantly, there were no differences in mortality between the intervention groups. Only one death in the EVAC group was reported. In the REBOA group, there was a restoration of SV, EF, ESV, EDV, and EDP and significant increases in SW, HR, CO, Ea, and ESP when compared with baseline. On average, we observed approximately 53% variability in CO within the REBOA group. Conversely, animals receiving the EVAC intervention displayed no significant differences in cardiac function at T74 compared with baseline and had significantly less variability than the REBOA group. This contrast in variability not only means that it is difficult to ascertain the impact of REBOA on cardiac function (requiring larger sample sizes), but also shows that the REBOA intervention can lead to potentially damaging effects on the heart and its performance compared with EVAC.

From the above observations, we can conclude that both EVAC and REBOA interventions significantly impacted the cardiac response, albeit at different magnitudes. The SW, Ea, ESV, EDV, and ESP were significantly higher in the REBOA group than in all other groups and displayed a significant amount of variability. These same cardiac function indices were significantly lower in the EVAC group. These findings coupled with the reduced variability in cardiac performance suggest that EVAC intervention produces a more consistent physiologic response with fewer extreme outliers. Our observations resemble the results of a study conducted by Russo et al. (31), wherein full aortic occlusion was found to induce the MAP levels at hemodynamically extreme levels, while partial aortic occlusions resulted in MAP levels higher than those observed in baseline, but lower than those observed during full aortic occlusion. It is our understanding that the strategies of partial aortic occlusion or variable aortic occlusion do not restrict flow as much as REBOA and therefore have a more modest effect on the cardiac physiologic response. As such, there must be less variation in the vascular autoregulation of flow compared with complete REBOA where the renal arteries are triggered by the low oxygen supply. There has been a wide range of findings in research performed on the variability in cardiac factors between animals subjected to cardiovascular injury models. For example, Stonko et al. (38) observed similar trends in Ea and ESP during a similar hemorrhage model, wherein both values decreased significantly. In contrast, while we observed significant increases in both parameters during REBOA and EVAC compared with the baseline values, their observations imply a slight restoration of these parameters in the REBOA group compared with the baseline values. Furthermore, the partial occlusion averages for both parameters in their study were lower than those observed in baseline, which differs from our observations. This difference may be attributed to differences in the hemorrhage model, where we implemented a fixed-volume hemorrhage, and they performed a fixed-pressure hemorrhage. Another notable difference in our study can be noticed in the *E_es_*, where we observed significant increases in all groups, in complete contrast to Stonko et al. where a significant decrease was observed. Nevertheless, our findings at T74 (i.e., after 44 min of either REBOA or partial occlusion with EVAC use) were in agreement with the literature, such that animals subjected to REBOA had higher average end-systolic elastance (*E*_es_) than the EVAC group.

A major contribution from this study was the development of a user-driven program to analyze the P-V loop data both within animals and across groups. We were able to successfully extract raw P-V loop data, apply robust methods to clean P-V data, remove erroneous loops, and identity ESP and EDP time points to get a single-beat analysis. The use of Python and its flexible data structures, such as lists and data frames, allowed for dynamic data storage without prior memory allocation. This enabled data preservation and allowed the users to avoid errant data point removal due to arbitrary set points or array size criteria. In addition, because we had access to data taken pre-IVCO, during IVCO, and post-IVCO, we developed methods to quantify both load-dependent vs. load-independent measurements of cardiac function while also considering intra-animal variability and intra-group variability. This allowed us to reliably quantify ESPVR and EDPVR, providing a framework to better evaluate the impact of endovascular devices that impact heart loading conditions while preserving cardiac inotropy, such as the use of IVCO, which decreases preload, or the administration of phenylephrine, which increases afterload (39), while also being able to quantify load-dependent measures of cardiac activity using the same mathematical framework. An advantage that our algorithm presents is that it allows for calculation of parameters from P-V loops across treatment groups and across experimental time points without introducing variations in analysis. Our methodology to incorporate a beat-to-beat analysis with the instantaneous heart rate calculations is important in ensuring that isochronism exists in the analyzed P-V loops. This yields benefit as with the decline of preload; a consistent heart rate is needed to ensure isochronism before the neuro-humoral axis activates. An isochronism is generally observed when the left ventricular pump maintains a constant speed independent of the load; loops that deviate from this tend to do so as a function of the activation of the neuro-humoral axis, which induces deviations in heart rate, making the loops ineligible to be analyzed for load-independent parameters.

Furthermore, an isochronism is essential when attempting to quantify load-independent parameters (39–41), but from what was observed in our data, isochronic P-V loops are unlikely to exist in raw data obtained during longitudinal hemorrhagic and EHC studies as they alter loading conditions and induce heart rate variability. Rather, loops will be expected to differ in size and shape, revealing the dynamic changes in autonomic function (42). Our algorithm offers a systematic method to analyze irregular P-V loops and individual beat-to-beat variability in a robust manner, particularly within the context of hemorrhagic shock and aortic occlusion. This type of analysis can significantly improve our understanding of the acute and transient changes in cardiac performance due to hemorrhage and aortic occlusion, while also accounting for the existence of a number of other acute conditions such as exercise, cardiac arrest recovery, and septic shock (43–45).

### Limitations

While we were successful in analyzing the within-subject and within-group variability of cardiac metrics with our custom Python algorithm, we did face a few challenges and limitations. First, we cannot stress the importance of acquiring high quality P-V loop data. In our experience, during acute dynamic periods (e.g., hemorrhagic shock and/or IVCO), there is a need for re-calibration of the P-V loop to account for any blood resistivity and/or blood volume changes. In some instances, there is also a need to re-position the P-V loop catheter. Failure to conduct these steps can lead to errant P-V loops, which can yield little to no meaningful end-systolic and end-diastolic data. Therefore, there is a need to define a standardized operating procedure for both acquisition and analysis of P-V loop data. Better definition of isochronic P-V loops during IVCO can also lead to higher fidelity P-V analysis and enable better comparison of data gathered under different conditions. Second, when quantifying the ESPVR, we originally incorporated six possible curve fits for the ESPVR, based on the established literature detailed by Claessens et al. (40). Similarly, we also assessed six curve fits for the EDPVR, as described by Burkhoff et al. (39). In the paper, we reported the linear and polynomial fits for ESPVR and EDPVR, respectively. However, it is important to note that the linear fit may not have always resulted in the best fit. Further work is needed to determine the impact of non-linear (or curvilinear) ESPVR relationships within the context of hemorrhagic shock and aortic occlusion. Third, there were some challenges presented with mining the temporal data and longitudinal P-V loops due to the incidence of sporadic arrhythmias. These points were selected and removed within the framework of the algorithm, but in the future, there should be considerations of analyzing P-V loop data in tandem with ECG and come up with novel ways to incorporate the arrhythmic P-V loops. This may be beneficial when comparing the incidence of arrhythmias in response to hemorrhage and/or endovascular hemorrhage control interventions. Finally, this study focuses predominantly on the cardiac metrics of systolic function. Currently, there is sparse research investigating cardiac performance during diastole in response to hemorrhage and resuscitation, and consequently a less comprehensive understanding of myocardial perfusion. Recent studies by Stonko et al. and Elansary et al. have started to uncover this relationship between LV function and coronary flow. Stonko et al. (38) first reported coronary artery measurements in response to REBOA and found that the total coronary flows were almost three times higher during a full aortic occlusion than baseline, and subsequently decreased with a partial occlusion, proportional to increases in *E_es_*. Then, Elansary et al. (46) examined the directionality of coronary artery flow waveform and LV function in progressive levels of hemorrhagic shock and found that the coronary arteries spent a greater proportion of diastole in retrograde flow. The findings from these studies indicate that future investigations incorporating both LV function and coronary flow would lead to a more holistic understanding of the underlying cardiovascular physiology of hemorrhagic shock and EHC interventions.

## Conclusion

In this study, we successfully developed an algorithm to perform a beat-to-beat analysis of P-V loops under both load-independent and load-dependent conditions. We were able to quantify both individual variability within each subject at each time point and determine the group variability over time. Using our custom algorithm, we evaluated the effects of EVAC and REBOA interventions on cardiac function and found that the use of REBOA was associated with significantly increased variability and worse cardiac performance. These data suggest that EVAC and partial aortic occlusion strategies are associated with less drastic changes in cardiac functional metrics. Further studies are needed to determine the relationship between varying degrees of partial occlusion and cardiac performance at milder and more severe hemorrhage conditions.

## Data Availability

The raw data supporting the conclusions of this article will be made available by the authors, without undue reservation.
