# Supplementary material for: Investigating the variability in pressure–volume relationships during hemorrhage and aortic occlusion
Source: Front Cardiovasc Med. 2023 Aug 23;10:1171904. doi: 10.3389/fcvm.2023.1171904 (PMC10482261; doi:10.3389/fcvm.2023.1171904)
Supplement: Supplementary file 1 [file Datasheet1.docx]

# Supplementary File

**List of Abbreviations**

**CO** - Cardiac Output

**Ea** - arterial elastance

**EDPVR** - End-diastolic pressure volume relation

**EF** - ejection fraction

**EHC** - endovascular hemorrhage control

**ESP** - End-systolic pressures

**ESPVR** - End-systolic pressure volume relation

**ESV** - End-systolic volumes

**EVAC** - Endovascular variable aortic control

**HR** - Heart Rate

**IVCO** - Inferior vena cava occlusion

**MAP** - mean arterial pressure

**NCTH** - non-compressible truncal hemorrhage

**P-V** - Pressure-volume

**REBOA** - Resuscitative Endovascular Balloon of the Aorta

**SV** - Stroke Volume

**SW** - Stroke Work

**Supplemental Figure 1. Illustration of P-V loop analysis by quadrants.** ) Four key events in the
cardiac cycle are defined by the pressure gradient (i.e., dP/dt max and dP/dt min). i) Aortic valve
opening (turquoise), ii) End of systole (red), iii) End of isovolumetric relaxation (pink), and iv) end
of diastole (purple), B) Four key elements of the cardiac cycle derived from the timepoints defined in
Figure 1A. This includes the i) Ejection Phase (blue), ii) Isovolumetric Relaxation (red), iii) Left Ventricular filling (purple) and iv) Isovolumetric contraction (turquoise).

| **A.**  **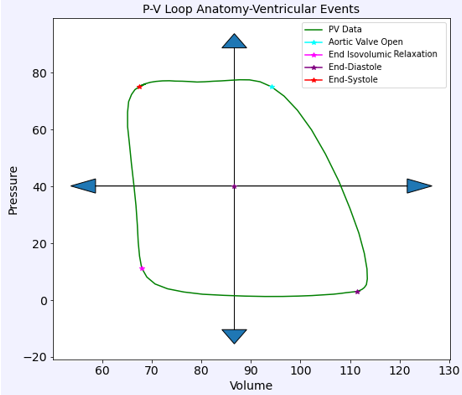** | **B.**  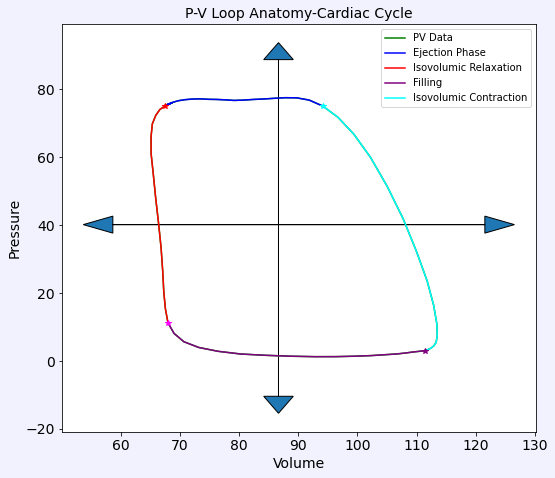 |
| --- | --- |

**Supplemental Figure 2. Sample Algorithm Results.** A) Sample of Pre-Screening Report, B) Sample Parameter Histogram, C) Sample Parameter Beat-to-Beat Report, D) Sample Parameter Time Variation Report

| **A.**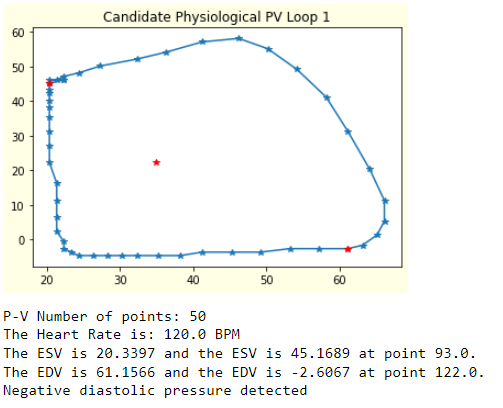 | **B.**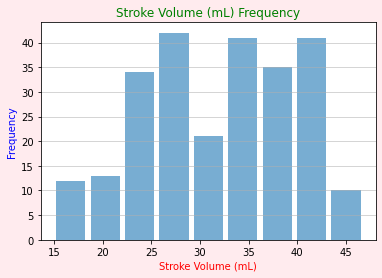 |
| --- | --- |
| **C.**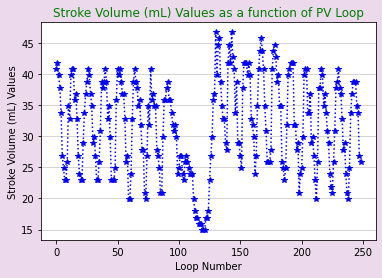 | **D.**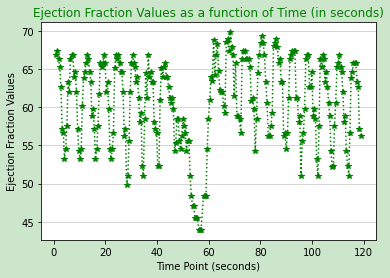 |

**Supplemental Table 1. Load-Independent Curve Fits.** To address varying curve fits for the EDPVR and ESPVR our code has incorporated six possible curve fits for both. However, for the purpose of the manuscript we focused on the linear curve fit for ESPVR as described the methods.

| EDPVR Curve Fits | ESPVR Curve Fits |
| --- | --- |
| $P=A+Be^{\alpha V}$  $P=Ce^{\beta V}$  $P=D+aV^{3}$  $P=a_{0}+a_{1}V+a_{2}V^{2}+a_{3}V^{3}$  $P=b+cV^{\gamma}$  $P=dV^{\delta}$ | $P=a_{1}V+a_{0}$  $P=a_{1}V+a_{0}, where P\left( V_{0,lin} \right)=0$  $P=a_{2}V^{2}+a_{2}V+a_{0}$  $P=a_{2}V^{2}+a_{2}V+a_{0}, where P\left( V_{0,quad} \right)=0$  $P=\ln\left( \frac{V}{V_{0}} \right)\left( \alpha+\beta V \right)^{-1}$  $P=\ln\left( \frac{V}{V_{0}} \right)\left( \alpha+\beta V \right)^{-1}, where P\left( V_{0,log} \right)=0$ |

**Supplemental Figure 3. Representative ensemble average P-V loops at baseline. A) Pre-IVCO, B) During-IVCO, C) Post-IVCO and D) Cumulative P-V loops are illustrated.** One minute of data was extracted and processed to calculate the ensemble average, except for during IVCO phases, where we had ~15 seconds of data. The ensemble average provides a singular representation of the left ventricular P-V relationship during each phase as opposed to selecting an individual loop which may have been impacted by a respiration artifact, allowing for comparison of P-V events pre-, during- and post-IVCO to be reasonably compared. As seen below, during IVCO, the P-V loops experience a reduction in preload, which translates to reduced pressure and volumes and thus a relatively smaller “loop” size. Post-IVCO the P-V loop returns to the pre-IVCO state. This shift occurs consistently throughout our experiment.

| **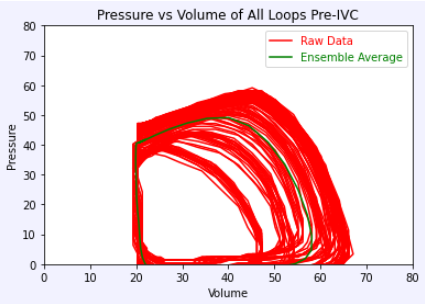**  **A) Ensemble average super-imposed to data sampled Pre-IVCO** | **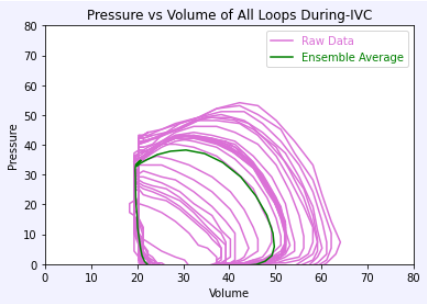**  **B) Ensemble average super-imposed to data sample during IVCO** |
| --- | --- |
| **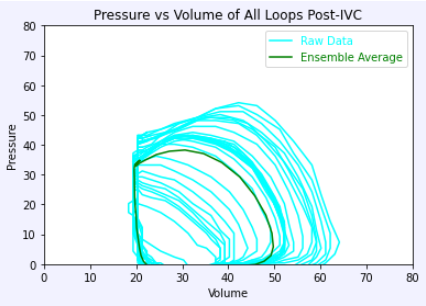**  **C) Ensemble average super-imposed to data sampled Post-IVCO** | **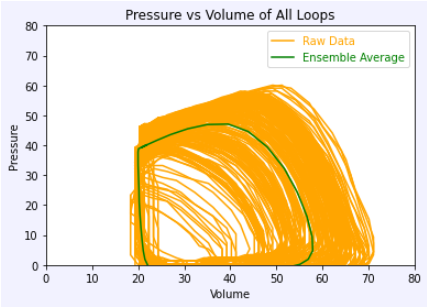**  **D) Ensemble average super-imposed to data sampled Pre-, During- and Post-IVCO** |

**Supplemental Table 2. Summary Curve Fit Parameters for EDPVR During IVCO.** Mean and standard deviations for each parameter at baseline (T0), end of hemorrhage (T30) and at the end of intervention (T74) are reported. A least squares regression for best fit was used to determine D and a curve fit parameters.

|  |  | **EDPVR**  $\boldsymbol{P}_{\boldsymbol{ed}}\boldsymbol{=D+a}{\boldsymbol{V}_{\boldsymbol{ed}}}^{\boldsymbol{3}}$ | |
| --- | --- | --- | --- |
| **Time Point** | **Group** | ***D***  ***mmHg*** | ***a***  ***mmHg/***$\boldsymbol{mL}^{\boldsymbol{3}}$ |
| **T0**  **Baseline** | **Control** | 0.54±4.57 | 9.44E-6 ± 5.92E-6 |
|  | **REBOA** | 3.09±4.07 | 3.28E-6 ± 5.10E-6 |
|  | **EVAC** | 3.41±5.89 | -2.49E-6 ± 1.09E-6 |
| **T30**  **End of Hemorrhage** | **Control** | 2.46±4.47 | 2.69E-5 ± 3.06E-5 |
|  | **REBOA** | 4.4±0.47 | 1.05E-5 ± 1.58E-5 |
|  | **EVAC** | 1.68±2.07 | 0.47E-5 ± 1.01E-5 |
| **T74**  **End of Intervention** | **Control** | 1.82±1.41 | 6.24E-6 ± 3.62E-6 |
|  | **REBOA** | 1.1±2.94 | 9.78E-6 ± 2.89E-6 |
|  | **EVAC** | 3.55±4.49 | 2.77E-6 ± 6.38E-6 |

| **Supplemental Table 3A. Summary of Control Group Cardiac Metrics.** Mean and standard deviations are reported at each time point and pre-IVCO, during IVCO and post-IVCO. | | | | | | | | | |
| --- | --- | --- | --- | --- | --- | --- | --- | --- | --- |
|  | **T0**  **Pre-IVCO** | **T0 During IVCO** | **T0**  **Post-IVCO** | **T30**  **Pre- IVCO** | **T30**  **During IVCO** | **T30**  **Post- IVCO** | **T74**  **Pre- IVCO** | **T74**  **During IVCO** | **T74**  **Post- IVCO** |
| **Stroke Work** (mmHg*mL) | 3300.8 ± 651.9 | 1973.6 ± 681.3 | 3190.5 ± 619.8 | 1451.5 ± 636.3 | 742.5 ± 344.3 | 1686.3 ± 718.4 | 4226.4 ± 801.8 | 2107.8 ± 653.2 | 4152.3 ± 1103.7 |
| **Stroke Volume** (mL) | 44.6 ± 10.8 | 35.5 ± 10.1 | 43.7 ± 11.8 | 38.6 ± 10.9 | 25.3 ± 7.6 | 41.1 ± 12.2 | 55.9 ± 9.2 | 41.8 ± 4.4 | 51.7 ± 11.0 |
| **Ejection Fraction** (%) | 42.2 ± 10.9 | 47.6 ± 13.7 | 41.9 ± 12.1 | 59.0 ± 10.6 | 49.5 ± 8.5 | 61.7 ± 9.7 | 53.8 ± 5.9 | 54.4 ± 6.5 | 51.9 ± 8.0 |
| **Heart Rate** (bpm) | 91.3 ± 19.5 | 92.4 ± 19.6 | 91.9 ± 20.2 | 127.7 ± 34.6 | 123.9 ± 29.6 | 119.5 ± 26.0 | 136.5 ± 19.9 | 136.1 ± 21.6 | 133.0 ± 24.1 |
| **Cardiac Output** (L/min) | 4.0 ± 1.1 | 3.3 ± 1.1 | 4.0 ± 1.2 | 5.1 ± 2.5 | 3.3 ± 1.7 | 5.1 ± 2.4 | 7.7 ± 1.9 | 5.7 ± 0.9 | 6.9 ± 1.9 |
| **Arterial Elastance** (mmHg/mL) | 1.8 ± 0.5 | 1.7 ± 0.4 | 1.9 ± 0.8 | 1.1 ± 0.4 | 1.4 ± 0.4 | 1.0 ± 0.3 | 1.4 ± 0.5 | 1.4 ± 0.4 | 1.5 ± 0.5 |
| **ESV** (mL) | 63.3 ± 17.7 | 42.9 ± 20.4 | 63.0 ± 19.2 | 26.6 ± 6.4 | 25.5 ± 5.8 | 25.2 ± 5.5 | 48.4 ± 5.9 | 34.6 ± 8.3 | 48.6 ± 9.5 |
| **ESP** (mmHg) | 74.9 ± 7.5 | 56.7 ± 5.7 | 72.7 ± 8.0 | 37.4 ± 7.7 | 32.2 ± 5.4 | 39.0 ± 7.7 | 71.6 ± 15.8 | 53.4 ± 10.5 | 72.9 ± 14.5 |
| **EDV** (mL) | 107.9 ± 13.1 | 78.3 ± 22.2 | 106.7 ± 13.7 | 65.2 ± 11.1 | 50.7 ± 10.5 | 66.3 ± 13.4 | 104.3 ± 9.3 | 76.4 ± 8.8 | 100.3 ± 11.5 |
| **EDP** (mmHg) | 6.7 ± 2.3 | 4.8 ± 3.6 | 6.4 ± 2.7 | 4.4 ± 2 | 3.8 ± 2.4 | 3.2 ± 2.4 | 7.2 ± 2.0 | 5.6 ± 0.8 | 4.2 ± 1.6 |

| **Supplemental Table 3B. Summary of REBOA Group Cardiac Metrics.** Mean and standard deviations are reported at each time point and pre-IVCO, during IVCO and post-IVCO. | | | | | | | | | |
| --- | --- | --- | --- | --- | --- | --- | --- | --- | --- |
|  | **T0**  **Pre-IVCO** | **T0 During IVCO** | **T0**  **Post-IVCO** | **T30**  **Pre- IVCO** | **T30**  **During IVCO** | **T30**  **Post- IVCO** | **T74**  **Pre- IVCO** | **T74**  **During IVCO** | **T74**  **Post- IVCO** |
| **Stroke Work** (mmHg*mL) | 3647.9 ± 1097.7 | 1763.9 ± 636.4 | 3447.8 ± 1081.4 | 1823.5 ± 460.3 | 414.3 ± 151.2 | 1908.1 ± 423.9 | 9327.8 ± 3237.8 | 5680.8 ± 4349.5 | 8785.4 ± 3016.3 |
| **Stroke Volume** (mL) | 50.3 ± 20.7 | 36.1 ± 11.7 | 51.7 ± 18.7 | 46.2 ± 14.1 | 22.8 ± 5.1 | 47.9 ± 14.3 | 59.4 ± 33.2 | 49.2 ± 30.9 | 61.3 ± 29.6 |
| **Ejection Fraction** (%) | 41.8 ± 9.6 | 45.2 ± 8.7 | 45.6 ± 7.4 | 60.2 ± 8.4 | 39.5 ± 2.8 | 62.5 ± 5.5 | 45.2 ± 13.4 | 51.3 ± 14.9 | 49.5 ± 11.0 |
| **Heart Rate** (bpm) | 89.8 ± 14.6 | 92.9 ± 19.5 | 88.9 ± 13.5 | 117.5 ± 23.3 | 113.2 ± 19.2 | 111.2 ± 22.3 | 163.4 ± 16.5 | 154.9 ± 34.1 | 157.6 ± 17.4 |
| **Cardiac Output** (L/min) | 4.8 ± 2.9 | 3.5 ± 1.7 | 4.8 ± 2.6 | 5.5 ± 2.3 | 2.6 ± 0.9 | 5.4 ± 2.2 | 9.7 ± 5.6 | 7.8 ± 5.4 | 9.6 ± 4.8 |
| **Arterial Elastance** (mmHg/mL) | 1.7 ± 0.6 | 1.7 ± 0.6 | 1.4 ± 0.4 | 1.0 ± 0.4 | 1.4 ± 0.8 | 0.9 ± 0.4 | 3.9 ± 2.0 | 2.7 ± 1.1 | 3.0 ± 1.7 |
| **ESV** (mL) | 68.4 ± 15.7 | 44.3 ± 7.1 | 61.7 ± 17.0 | 30.9 ± 11.0 | 35.6 ± 12.2 | 29.0 ± 9.7 | 68.9 ± 21.1 | 43.2 ± 10.9 | 60.6 ± 14.5 |
| **ESP** (mmHg) | 72.1 ± 3.7 | 55.3 ± 8.8 | 66.2 ± 7.0 | 39.3 ± 7.8 | 28.6 ± 7.5 | 39.1 ± 8.7 | 173.8 ± 18.9 | 108.9 ± 42.2 | 143.0 ± 28.8 |
| **EDV** (mL) | 118.7 ± 27.0 | 80.4 ± 11.8 | 113.4 ± 29.6 | 77.1 ± 21.3 | 58.4 ± 17.3 | 76.9 ± 22.0 | 128.3 ± 43.3 | 92.4 ± 33.7 | 121.9 ± 36.1 |
| **EDP** (mmHg) | 6.6 ± 3.5 | 4.8 ± 3.7 | 6.1 ± 2.8 | 2.4 ± 1.8 | 3.5 ± 2.2 | 2.3 ± 1.6 | 6.4 ± 1.6 | 5.3 ± 2.8 | 5.7 ± 1.9 |

| **Supplemental Table 3C. Summary of EVAC Group Cardiac Metrics.** Mean and standard deviations are reported at each time point and pre-IVCO, during IVCO and post-IVCO. | | | | | | | | | |
| --- | --- | --- | --- | --- | --- | --- | --- | --- | --- |
|  | **T0**  **Pre-IVCO** | **T0 During IVCO** | **T0**  **Post-IVCO** | **T30**  **Pre- IVCO** | **T30**  **During IVCO** | **T30**  **Post- IVCO** | **T74**  **Pre- IVCO** | **T74**  **During IVCO** | **T74**  **Post- IVCO** |
| **Stroke Work** (mmHg*mL) | 3201.4 ± 744.0 | 1994.0 ± 754.2 | 3132.3 ± 792.3 | 1840.7 ± 342.1 | 1236.4 ± 449.1 | 2530.2 ± 641.4 | 4771.3 ± 870.8 | 3017.5 ± 1454.4 | 4621.4 ± 871.9 |
| **Stroke Volume** (mL) | 42.9 ± 14.6 | 24.8 ± 5.1 | 42.7 ± 15.2 | 39.2 ± 5.8 | 29.5 ± 5.4 | 43.6 ± 9.3 | 34.2 ± 0.9 | 28.0 ± 4.7 | 34.3 ± 4.4 |
| **Ejection Fraction** (%) | 44.5 ± 12.9 | 38.5 ± 12.0 | 44.9 ± 13.8 | 62.2 ± 8.0 | 55.2 ± 10 | 64.3 ± 7.0 | 39.0 ± 6.08 | 41.1 ± 8.3 | 40.8 ± 7.7 |
| **Heart Rate** (bpm) | 88.5 ± 18.9 | 86.0 ± 22.2 | 85.5 ± 13.4 | 135.4 ± 31.1 | 133.7 ± 29.4 | 118.6 ± 21.8 | 135.05 ± 27.78 | 130.2 ± 30.4 | 135.4 ± 26.4 |
| **Cardiac Output** (L/min) | 3.8 ± 1.5 | 2.1 ± 0.4 | 3.7 ± 1.5 | 5.4 ± 1.8 | 3.9 ± 0.8 | 5.3 ± 1.8 | 4.6 ± 0.9 | 3.6 ± 0.9 | 4.6 ± 1.0 |
| **Arterial Elastance** (mmHg/mL) | 2.0 ± 0.8 | 2.9 ± 0.8 | 2.0 ± 0.8 | 1.2 ± 0.3 | 1.5 ± 0.3 | 1.3 ± 0.4 | 4.07 ± 0.69 | 3.6 ± 0.8 | 4.0 ± 1.3 |
| **ESV** (mL) | 55.3 ± 21.4 | 42.3 ± 17.9 | 54.2 ± 22.0 | 24.8 ± 8.7 | 24.2 ± 7.7 | 24.8 ± 8.1 | 55.65 ± 15.96 | 41.7 ± 11.1 | 51.9 ± 16.4 |
| **ESP** (mmHg) | 76.1 ± 5.1 | 64.8 ± 11.5 | 73.4 ± 7.5 | 46.7 ± 6.5 | 42.0 ± 7.3 | 53.4 ± 11.2 | 134.62 ± 22.43 | 99.3 ± 25.1 | 123.8 ± 21.4 |
| **EDV** (mL) | 98.2 ± 22.8 | 67.1 ± 18.8 | 96.9 ± 22.4 | 64.0 ± 11.7 | 53.8 ± 7.8 | 68.4 ± 14.8 | 89.8 ± 15.69 | 69.7 ± 9.3 | 86.2 ± 16.4 |
| **EDP** (mmHg) | 7.0 ± 3.4 | 4.5 ± 3.7 | 6.9 ± 3.5 | 5.1 ± 5.2 | 3.7 ± 4.2 | 2.9 ± 3.1 | 7.46 ± 4.04 | 5.1 ± 3.3 | 6.0 ± 4.6 |
